# Supplementary material for: Causal interplay between lactose intolerance and gut microbiota: a combined bidirectional Mendelian randomization and in vivo validation study
Source: Front Nutr. 2026 Jun 1;13:1803337. doi: 10.3389/fnut.2026.1803337 (PMC13265576; doi:10.3389/fnut.2026.1803337)
Supplement: Supplementary file 1 [file Data_Sheet_1.zip › supplementary materials/Forward/leave one out cross validation/ebi-a-GCST90027594.finngen_R12_E4_LACTONAS.pdf]

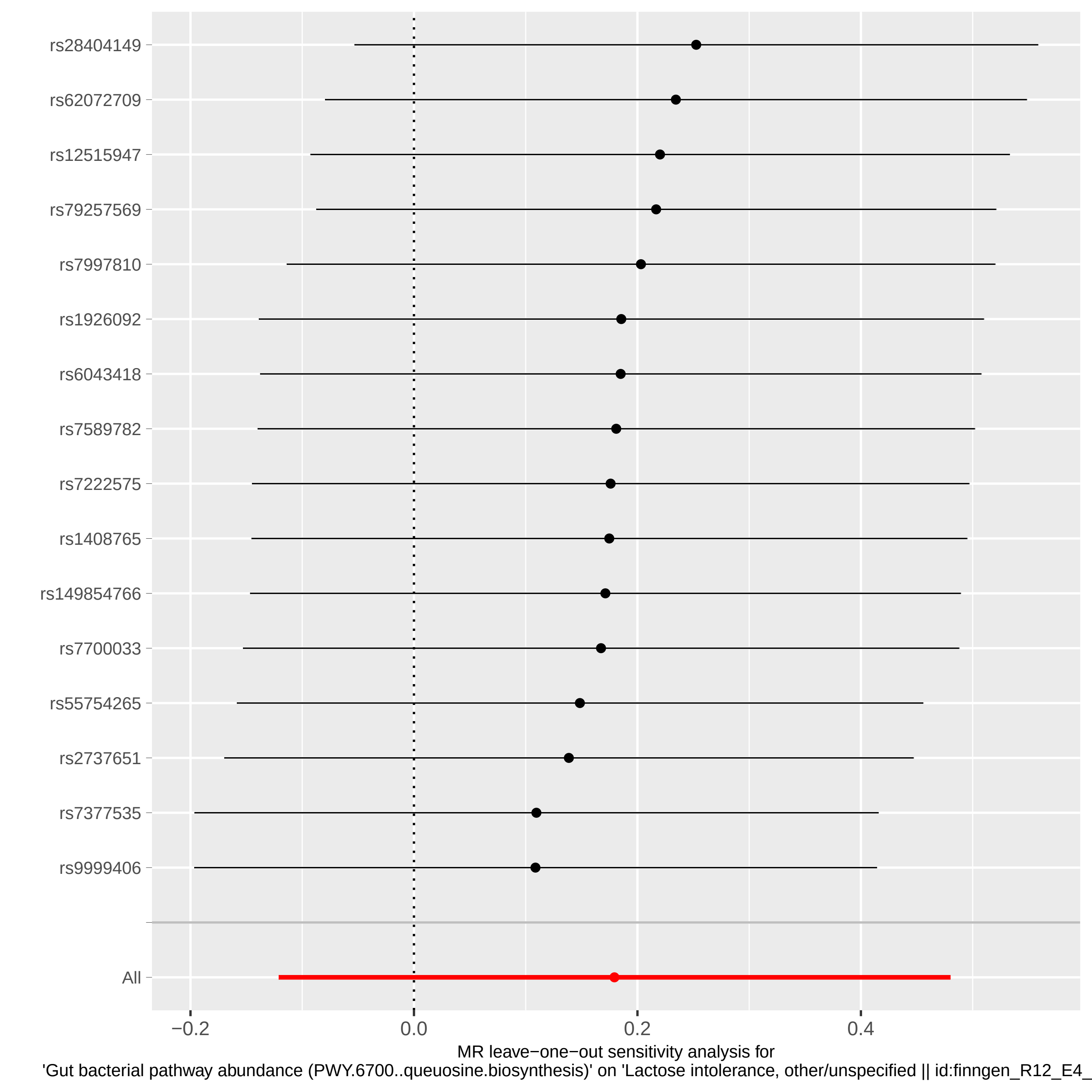
rs28404149
rs62072709
rs12515947
rs79257569
rs7997810
rs1926092
rs6043418
rs7589782
rs7222575
rs1408765
rs149854766
rs7700033
rs55754265
rs2737651
rs7377535
rs9999406
All
−0.2
0.0
0.2
0.4
MR leave−one−out sensitivity analysis for
'Gut bacterial pathway abundance (PWY.6700..queuosine.biosynthesis)' on 'Lactose intolerance, other/unspecified || id:finngen_R12_E4_
